# Dietary factors and low-grade inflammation in relation to overweight and obesity revisted

**DOI:** 10.1017/S0007114522000782

**Published:** 2022-05-28

**Authors:** Philip C. Calder

**Affiliations:** 1 School of Human Development and Health, Faculty of Medicine, University of Southampton, Southampton, UK email pcc@soton.ac.uk; 2 NIHR Southampton Biomedical Research Centre, University Hospital Southampton NHS Foundation Trust and University of Southampton, Southampton, UK

**Fig. 1.**
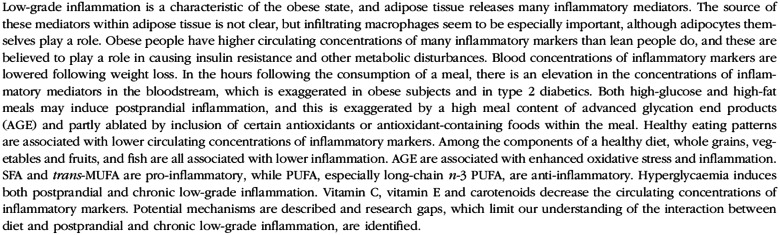
Abstract of Calder *et al.* ^(1)^

In 2011, the *British Journal of Nutrition* published the output of the work of an expert group assembled by the European Branch of the International Life Sciences Institute (ILSI Europe) with the aim of exploring the role of low-grade inflammation in overweight and obesity and identifying the potential of dietary exposures to modify that process^(1)^. The abstract of that publication is shown in Fig. 1. According to Web of Science, the paper has now been cited 561 times, being the second most cited paper published in the *British Journal of Nutrition* in 2011 and the 21st most cited of all papers ever published in the journal. Citations of the paper have been sustained over time, being between 43 and 64 per year over the period 2013 to 2019. Remarkably, the highest number of citations was received in 2020 and 2021 with 68 and 84 citations, respectively. The pattern of citations suggests a continued relevance of the paper, and the higher number in the last two years undoubtedly reflects the recognition of the contributions of both inflammation and overweight and obesity to poor outcome from coronavirus disease discovered in 2019 (COVID-19). This paper built on the activity of an earlier ILSI Europe expert group that considered biomarkers of inflammatory processes in different physiological and pathological states^(2)^ and related to later expert group activities that gave a deeper consideration to biomarkers of inflammation that might be used in the substantiation of health clams^(3)^ and to the role of low-grade inflammation in ageing and the potential of dietary exposures to modify that process^(4)^. Those papers are also fairly well cited with 204, 196 and 180 Web of Science citations, respectively, reflecting the enduring interest in inflammation as it relates to diet and nutrition and to different states and stages of human physiology.

Inflammation is a component of innate immune responses and, as such, is a normal mechanism involved in host defence against pathogenic organisms and other insults. Physiologically, inflammatory responses are self-regulating. Loss of such self-regulation is linked with many pathological states, where the on-going unregulated inflammatory responses cause damage to host tissues. The diseases that result involve activated inflammatory cells and excessive inflammatory mediator production at the site of tissue damage with elevated concentrations of markers of inflammation in the systemic circulation. The latter markers include acute phase proteins, such as C-reactive protein, and cytokines such as TNF and IL-6. Examples of such diseases include rheumatoid arthritis and the inflammatory bowel diseases. The impacts of these diseases are controlled, with varying degrees of success, with anti-inflammatory pharmaceutical agents. In the 1990s, it was discovered that adipose tissue can produce inflammatory cytokines^(5,6)^, and in the first decade of the 2000s, there were many reports that the circulating concentrations of inflammatory markers, including C-reactive protein, TNF and IL-6, are higher in individuals living with obesity than in age-and sex-matched healthy weight controls (e.g.^(7–9)^). This state of enhanced inflammation could link obesity with its co-morbidities like type-2 diabetes, metabolic fatty liver disease and CHD, in part because the inflammatory mediators could have secondary effects at other sites (e.g. the liver or the blood vessel wall) and in part because inflammation induces insulin resistance. The concentrations of inflammatory markers observed in those with obesity, though higher than in controls, were much lower than observed in individuals with frank inflammatory diseases. Hence, obesity came to be recognised as a state of low-grade inflammation, a term that has only been widely used in the last two decades (the oldest paper identified in a PubMed search using ‘Adipose tissue AND Low grade inflammation’ was published in 1999^(10)^ and this is the third oldest paper identified in a search using ‘Obesity AND Low grade inflammation’). Therefore, at the time of the work of the ILSI Europe expert group that was published in 2011, the broad recognition that obesity and inflammation are somehow linked was fairly new. In parallel with research on inflammation in obesity, was the research on the influence of many foods and nutrients on inflammatory processes, with some foods and nutrients apparently increasing inflammation and others dampening it. It had also been discovered that the gut microbiota appears to be altered in obesity^(11)^. Given that diet is a major determinant of the gut microbiota^(12)^ and that the gut microbiota may have a role in regulating inflammation^(13)^, there seem to be multiple axes of interaction between nutrition, the gut microbiota, adipose tissue and inflammation.

The ILSI Europe expert group set out to collate and review the evidence around obesity being a state of low-grade inflammation and the evidence for various diets and dietary components being modulators of inflammation. The paper begins with a discussion of the concept of low-grade inflammation and provides copious evidence from human research that obesity is a state of low-grade inflammation, based mainly on measurements made in blood. It goes on to describe adipose tissue as a source of inflammatory mediators, explains how both adipocytes and infiltrating inflammatory cells from blood (especially monocyte-derived macrophages) are sources of these and that the inflammatory milieu of the adipose tissue influences macrophage differentiation into phenotypes that are more or less inflammatory in nature. The evidence that visceral adipose tissue is ‘more inflammatory’ than subcutaneous is described and then the role of inflammation in modulating insulin signalling and insulin sensitivity is reviewed. The paper then moves on to nutritional aspects. The phenomenon of post-prandial inflammation is described: both high simple sugar and high fat meals induce a state of elevated inflammation in the hours following their consumption, and there is a view that this is part of the link between poor quality diets and increased risk of non-communicable diseases^(14)^. Inclusion of fibre, some plant polyphenolic compounds or *n*-3 fatty acids, amongst others, in the meal can partly mitigate its effects on inflammation. The paper goes on to review the effects of different eating patterns, whole foods and beverages, glycated end products, fatty acids, carbohydrates, milk peptides, vitamin D, antioxidant vitamins (C and E and carotenoids), flavonoids and phytoestrogens on inflammatory markers as reported in human studies, although often not in those with obesity. Finally, the paper descries the impact of an altered gut microbiota on inflammatory makers and the effects of pre and probiotics. As such, the paper provides a comprehensive overview of adipose tissue, obesity and inflammation and of nutrition and inflammation and attempts to integrate these. In this respect, the paper was unique at the time of its publication. This probably explains its sustained high level of citations over the 10 years since its publication. However, as noted earlier, citations have gone up during the period of the COVID-19 pandemic. Outcomes from COVID-19 are worse in those with higher inflammation^(15,16)^, are worse in those living with obesity^(17,18)^ and may be worse in those with poor nutrition^(19,20)^. Because the paper by Calder *et al*.^(1)^ brings obesity, inflammation and nutrition together, it remains an attractive paper to cite by those publishing about COVID-19.

In the ten years since the publication by Calder *et al*.^(1)^, research in the area of adipose tissue, obesity and inflammation has increased significantly (Table 1). Much more is known about inflammation within human adipose tissue including that visceral adipose tissue has a higher state of inflammation than subcutaneous^(21)^ and that infiltrating cells other than macrophages, and including dendritic cells, T cells and B cells, make important contributions to adipose tissue inflammation^(22)^. There are interesting studies reporting altered concentrations of recently discovered *n*-3 fatty acid-derived lipid mediators that act to resolve (‘turn off’) inflammation in human adipose tissue^(23)^, suggesting a nutritional strategy that could reduce adipose tissue inflammation with the aim of mitigating some of the co-morbidities associated with obesity. Earlier studies reported that *n*-3 fatty acids (EPA + DHA) could decrease macrophage numbers, crown-like structures and expression of some inflammatory genes in human subcutaneous adipose tissue^(24,25)^ and could increase concentrations of pro-resolving lipid mediators mainly in visceral adipose tissue^(25)^. A more recent study reported that *n*-3 fatty acids could alter endocannabinoid and other lipid mediator concentrations and gene expression in human subcutaneous adipose tissue but that adipose tissue from those living with obesity showed less profound changes than that from healthy weight individuals^(26,27)^. This study has raised questions about better targeting of adipose tissue in those living with obesity. Against this background of advances in our understanding of adipose tissue biology, of obesity as a state of low-grade inflammation and of nutritional strategies to reduce the inflammatory state of adipose tissue, the paper by Calder *et al*.^(1)^ will remain relevant for some time and seems likely to continue to be cited.

**Table 1.** Numbers of publications identified in PubMed using different search terms. Searches conducted 27 February 2022

	Search terms used			
Years covered	Obesity AND inflammation	Adipose tissue AND inflammation	Obesity AND low-grade inflammation	Adipose tissue AND low-grade inflammation
1980–1989	65	89	1	0
1990–1999	159	190	3	1
2000–2009	3961	1892	473	232
2010–2019	19 801	9940	2301	1156
2020-now	7433	3272	809	334
